# Extubation in the pediatric intensive care unit: predictive methods. An integrative literature review

**DOI:** 10.5935/0103-507X.20210039

**Published:** 2021

**Authors:** Jéssica Cristina da Silva Moura, Lívea Gianfrancesco, Tiago Henrique de Souza, Taís Daiene Russo Hortencio, Roberto José Negrão Nogueira

**Affiliations:** 1 Universidade Estadual de Campinas - Campinas (SP), Brazil.; 2 Faculdade de Medicina São Leopoldo Mandic - Campinas (SP), Brazil.

**Keywords:** Weaning respirator, Respiration, artificial, Airway extubation, Child, Desmame do respirador, Respiração artificial, Extubação, Criança

## Abstract

For extubation in pediatric patients, the evaluation of readiness is strongly recommended. However, a device or practice that is superior to clinical judgment has not yet been accurately determined. Thus, it is important to conduct a review on the techniques of choice in clinical practice to predict extubation failure in pediatric patients. Based on a search in the PubMed®, *Biblioteca Virtual em Saúde*, Cochrane Library and Scopus databases, we conducted a survey of the predictive variables of extubation failure most commonly used in clinical practice in pediatric patients. Of the eight predictors described, the three most commonly used were the spontaneous breathing test, the rapid shallow breathing index and maximum inspiratory pressure. Although the disparity of the data presented in the studies prevented statistical treatment, it was still possible to describe and analyze the performance of these tests.

## Abstract (pt)

Para a extubação orotraqueal em pacientes pediátricos, é fortemente recomendada a avaliação de sua prontidão. No entanto, a utilização de um dispositivo ou prática que fosse superior ao julgamento clínico ainda não foi determinada com exatidão. Assim, é importante realizar uma revisão sobre as técnicas preditoras de escolha na prática clínica para prever a falha de extubação orotraqueal em pacientes pediátricos. A partir de uma busca nas bases de dados PubMed®, Biblioteca Virtual em Saúde, *Cochrane Library* e Scopus, realizamos um levantamento das variáveis preditoras de falha de extubação orotraqueal mais comumente utilizadas na prática clínica em pacientes pediátricos. Dos oito preditores descritos, observamos três mais usados: teste de respiração espontânea, índice de respiração rápida e superficial e pressão inspiratória máxima. Embora a disparidade dos dados apresentados nos estudos tenha inviabilizado um tratamento estatístico, foi possível, a partir desse meio, descrever e analisar o desempenho desses testes.

## INTRODUCTION

Approximately 55% of children admitted to a pediatric intensive care unit (ICU) require mechanical ventilation (MV),^(1,2)^ and the intubation and extubation (EXT) of these patients are high risk and may be associated with increased morbidity and mortality.^(3)^ Several factors are related to this increase, such as the necessary ventilatory variables and the duration of MV.^(4)^ Despite the benefits of MV when properly indicated, its prolonged use can cause airway injuries, pulmonary infections, cardiovascular instability and complications resulting from immobility.^(5,6)^ Similarly, premature EXT can also be harmful because failure and the need for reintubation are associated with longer length of stay and cardiorespiratory and/or neurological impairments, which can result in long-term disability.^(7-9)^

According to the latest update of the Brazilian Guidelines for Mechanical Ventilation,^(10)^ EXT failure is defined as the need for reintubation within 48 hours after removal of the artificial airway. In the pediatric population, it is estimated that the failure rate ranges from 16% to 22%.^(11)^

The choice of the ideal time for EXT is a challenge and is usually made based on clinical judgment, based on the cardiorespiratory, neurological and hemodynamic status of the patient.^(12)^ Therefore, the use of a well-defined EXT readiness protocol is essential.^(13)^ The Pediatric Acute Respiratory Distress Syndrome: Consensus (PARDS)^(14)^ recommends the performance of daily evaluations of EXT readiness in pediatric patients, and the benefits of such evaluations have already been reported in the literature.^(1,15,16)^

Until 2017, the Recommendations for Mechanical Ventilation of Critically Ill Children from the Paediatric Mechanical Ventilation Consensus Conference did not have consistent data that indicated the use of a device or test to predict failure that was superior to a clinical evaluation. Thus, there was no recommendation of any specific method to establish EXT readiness.^(17)^

The objective of this study was to identify the predictors of choice in clinical practice to predict the success or failure of EXT in pediatric patients.

## METHODS

This was an integrative literature review; it includes an analysis of relevant research that supports decision-making and improvements in clinical practices. Thus, it allows summarizing the state of the art of the subject, in addition to identifying the knowledge gaps that need to be filled by new studies.^(18)^

Thus, the present study was conducted in the following steps: definition of the guiding question of the study, search, data extraction, analysis and synthesis of the results and data presentation.^(19)^

The guiding question for the present review was “What are the predictive methods of choice in clinical practice to predict the success or failure of EXT in pediatric patients?”. To answer this question, a search was conducted in the PubMed®, *Biblioteca Virtual em Saúde* (BVS), Cochrane Library and Scopus databases, from October 1, 2018, to October 31, 2018. The Medical Subject Headings (MeSH) “desmame do respirador”, “respiração artificial”, “extubação” and “pediatria”, in addition to their synonyms in Portuguese, and their English counterparts (“ventilator weaning”, “respiration, artificial”, “extubation”, and “pediatrics”), in addition to their respective synonyms, were used and combined using the Boolean operators *AND* and *OR* (Supplementary material). The present study was registered in the International Prospective Register of Systematic Reviews (PROSPERO) under identification CRD42019122207.

The studies included were clinical trials (randomized or not) with longitudinal designs comparing different techniques for the evaluation of indications for EXT in the pediatric population. Articles in languages other than Portuguese or English that did not have a well-defined EXT protocol and studies conducted exclusively in neonates were excluded. In addition, studies that did not comply with the definition of failure provided by the Brazilian Guidelines for Mechanical Ventilation,^(10)^ i.e., reintubation within 48 hours, were excluded.

Two reviewers independently evaluated all articles retrieved in the search and excluded duplicate references using Mendeley software (Mendeley Desktop©, Version 1.19.4, 2008 Glyph & Cog, LLC). Next, articles that did not meet the eligibility criteria were excluded. At this stage, when comparing the search results, any differences between the reviewers were resolved by consulting a third author. A manual search was also performed in the bibliographic references of the included articles.

To better understand the nature of the publications, a data collection instrument was developed, in which the title, publication journal, year of publication, authors, sample characteristics (sample size, age, and diagnosis), study design, weaning techniques, EXT failure rate and objectives were recorded.

**Figure 1:**
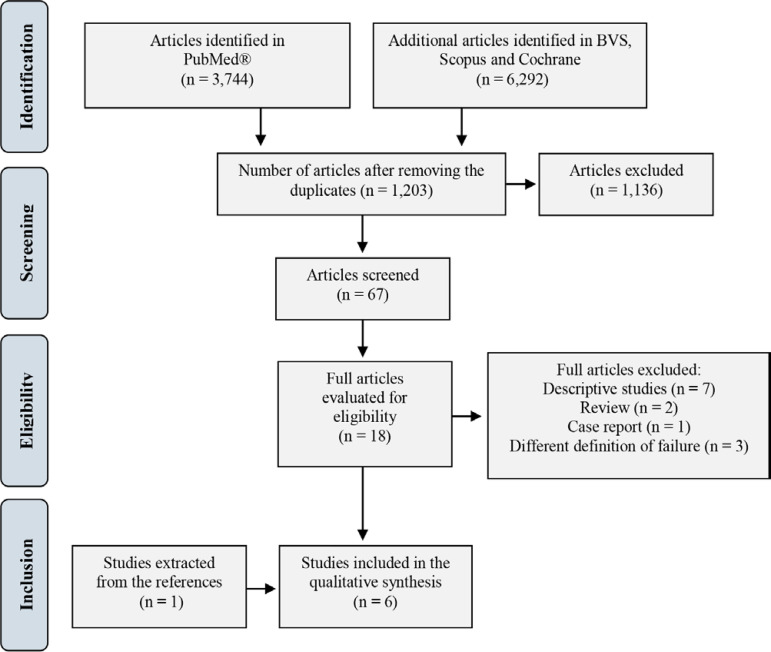
Flowchart, based on PRISMA, for study selection. BVS - Biblioteca Virtual em Saúde.

After the search, 10,036 articles were retrieved, and of these, 8,833 were excluded by assessing duplicates, 1,136 by reviewing the title and 49 by reading the abstract. After reading the abstracts, seven were excluded because they were descriptive studies, one because it was a case report, two because they were reviews and three because they did not use the definition of EXT failure established for our study. After reading the full articles and reviewing the references, a new study that met our criteria was included. Thus, six studies were included in this review. The phases of the selection process are summarized and presented in a flowchart (Figure 1), as recommended by the PRISMA group.^(20)^ After reading each of the selected articles, the following aspects were summarized: authors, country where the study was conducted, study design, number of participants, age, cause of intubation, chosen predictor and study objective.

## RESULTS

Regarding the design of the studies included in this review, two were prospective cohorts,^(21,22)^ two were clinical trials^(23,24)^ and two were randomized clinical trials.^(25,26)^ Table 1 presents a summary of these articles, with information about the general characteristics of the studies: study design, sample size, cause of intubation and objective of the study. Table 2 provides the predictors used and most relevant results from each study.

**Table 1:** Summary of publications included in the integrative review

References	Country	Study design	Number of participants	Age	Cause of intubation	Objective
Laham et al.^(21)^	United States	Prospective cohort	319	Not specified	42% clinical 58% surgical	To evaluate the practice of determining EXT readiness based on pre-EXT medical judgment
Khemani et al.^(22)^	United States	Prospective cohort	409	< 18 years	48.9% clinical 49.1% surgical 2% other	To identify risk factors for pediatric EXT failure
Riou et al.^(23)^	France	Clinical trial	42	1 month to 15.9 years	83.3% clinical 14.2% surgical 2.3% trauma	To evaluate VD/VT as a predictor of EXT failure
Johnston et al.^(24)^	Brazil	Clinical trial	40	≤ 12 months	100% clinical	To evaluate the performance of an EXT readiness test based on the SBT using PS
Foronda et al.^(25)^	Brazil	Randomized clinical trial	294	28 days to 15 years	100% clinical	To evaluate whether the combination of daily evaluations and the SBT can shorten the duration of MV compared to weaning based on a standard of care
Jouvet et al.^(26)^	Canada	Randomized clinical trial	30	2 to 18 years	40% clinical 40% surgical 20% trauma	To compare the duration of automated MV weaning *versus* usual weaning

EXT - extubation; VD/VT - dead space/tidal volume; SBT - spontaneous breathing test; MV - mechanical ventilation.

**Table 2:** Predictors used and main results of the studies included in the integrative review

References	Predictors used	Main results
Laham et al.^(21)^	SBT	The success rate in the first attempt at EXT was 91%. The risk factors associated with failure were duration of MV (OR = 2.20; p < 0.0001), pre-EXT corticosteroids (OR = 2.4; p = 0.04) and post-EXT stridor (OR) = 3.4; p < 0.01). Ventilation index ≤ 8 was associated with failure in a patient with 1 day of MV. EXT failure was associated with longer length of ICU stay and increased hospital costs; patients who failed stayed in the ICU 14 days longer (p < 0.0001), with a cost 3.2 times higher (p < 0.0001) than that incurred by patients with successful EXT
Khemani et al.^(22)^	PiMax, PI/PiMax, RSBI, TTI	Within 48 hours after EXT, 8.3% of patients were reintubated. The risk factors for reintubation included lower PiMax, longer MV duration, UAO post-EXT, high post-EXT respiratory effort (PRP and TTI) and high post-EXT phase angle. Approximately 35% of the children had a PiMax < 30cmH2O at the time of EXT and were almost three times more likely to be reintubated than those with a PiMax > 30cmH_2_O (p = 0.006). The reintubation rate was higher in children with a PiMax < 30cmH_2_O and PRP > 1,000. In children who developed UAO, the reintubation rate was higher in those with a PiMax < 30cmH_2_0 than in those with a PiMax > 30cmH_2_O (47% *versus* 15.4%; p = 0.02).
Riou et al.^(23)^	VD/VT	NIV was used in four patients who developed RF after EXT; there was no reintubation. Children who required NIV had significantly higher VD/VT than those who did not undergo NIV (p < 0.001). The cutoff value for VD/VT was 0.55, and the area under the ROC curve was 0.86.
Johnston et al.^(24)^	PiMax, RSBI, load/force balance	EXT failure occurred in 15% of extubated children. There were no significant differences in blood gas values or MV parameters between the EXT success and failure groups. There was a statistically significant difference between the groups for two risk factors: weight < 4kg and TV < 4mL/kg. The variables with a large area under the curve were minute volume < 0.8mL/kg/minute and PiMax < 50cmH_2_O. The variables with a small area under the curve were load/force balance > 5 and RSBI > 6.7.
Foronda et al.^(25)^	SBT	The MV duration was shorter in the test group (3.5 days) than in the control group (4.6 days) (p = 0.0127 (95%CI)). This significant reduction in the test group was not associated with an increase in the EXT failure rate or the use of NIV post-EXT. It represents a 30% reduction in the risk of remaining on MV (risk rate of 0.7)
Jouvet et al.^(26)^	SmartCare™	The median weaning duration was 21 hours (range, 3 - 142 hours) in the SmartCare™ group and 90 hours (range 4 - 552 hours) in the usual weaning group (p = 0.007). The reintubation rates and the use of NIV post-EXT with SmartCare™ and in the usual weaning group were 2/15 *versus* 1/15 and 2/15 *versus* 2/15, respectively.

SBT - spontaneous breathing test; EXT - extubation; MV - mechanical ventilation; OR - odds ratio; ICU - intensive care unit; PiMax - maximum inspiratory pressure; PI - esophageal pressure; SSRI - rapid shallow breathing index; TTI - tension-time index; UAO - upper airway obstruction; PRP - pressure rate product; VD/VT - dead space/tidal volume; NIV - noninvasive ventilation; RF - respiratory failure; ROC - receiver operating characteristic; VT - tidal volume; 95%CI - 95% confidence interval.

In total, 1,134 children were evaluated, 405 (35.7%) of whom were male. Only one study^(22)^ did not provide the sex of the participants. There was great variation in the size of the samples studied (n = 30 - 409). The age of patients included in the studies ranged from 28 days to 18 years; however, it was not possible to measure age as a common variable because of the differences in data presentation among the studies.

In the six studies, eight means of predicting EXT were identified: the spontaneous breathing test (SBT),^(21,25)^ the ratio of dead space to tidal volume (VD/VT),^(23)^ the rapid shallow breathing index (RSBI),^(22,24)^ maximum inspiratory pressure (PiMax),^(22,24)^ the ratio of esophageal pressure to PiMax (PI/PiMax),^(22)^ the tension-time index (TTI),^(22)^ load/force balance^(24)^ and an automated weaning protocol.^(26)^ The most commonly used methods to predict EXT failure were the SBT (2/6), the RSBI (2/6) and PiMax (2/6).

**Figure 2:**
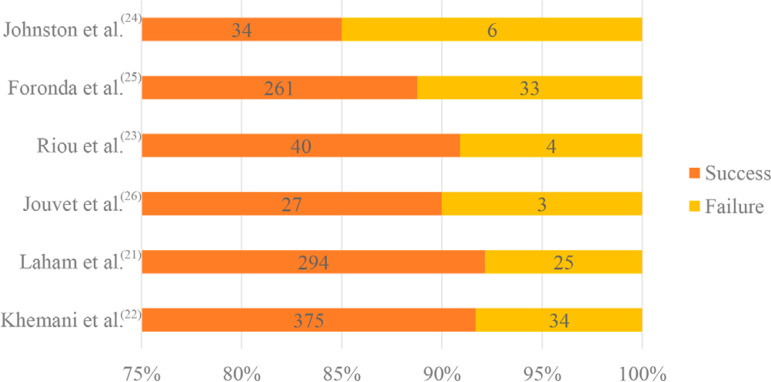
Extubation success and failure rates reported by the studies included in the integrative review.

The reason for intubation was mostly clinical. Of the 1,134 patients included in the review, 129 were reintubated, i.e., the success rate was 91.2%, and failure rate was 8.7% (Figure 2).

## DISCUSSION

The present study identified predictors of EXT, among which the most frequently used tools to predict EXT failure were the SBT, the RSBI and PiMax. The least commonly used methods were PI/PiMax, VD/VT, the TTI, load/force balance and automated weaning.

### Spontaneous breathing test

The SBT, previously traditionally used in adults, has been applied in pediatric patients, without specific adaptations for this population.^(27)^ It evaluates the patient’s ability to maintain spontaneous breathing by means of pressure support ventilation (PS), continuous positive airway pressure (CPAP), or a T-piece with oxygen delivery for 30 minutes to 2 hours.^(26)^ In this review, two studies associated the performance of the test with the EXT outcome, and their results differed.

Foronda et al.,^(25)^ when implementing a daily SBT protocol in their patients, with a PS of 10cmH_2_O for 2 hours, observed a reduction in the duration of MV in children, without increasing the EXT failure rate or need for noninvasive ventilation (NIV), as previously described in adults. However, SBT is performed with some caveats in pediatric clinical practice. It is speculated that the smaller diameter of the endotracheal tube (ETT) used in these patients increases respiratory work due to the increase in airway resistance. However, the inspiratory resistance in children is already physiologically high (approximately 80 - 90cmH_2_O/L/minute), while with ETT, the resistance is incorporated into the existing high flows (approximately 15 - 20cmH_2_O/L/minute), i.e., the ETT resistance becomes irrelevant.^(6)^ In addition, there is a new generation of VMs that are programmed to automatically compensate for ETT resistance.

In a study by Laham et al.,^(21)^ SBT was performed with PS or CPAP that maintained a VT between 5 and 7cm^3^/kg. It was performed at the medical discretion of 70% of patients. In this case, the clinical judgment in decision-making showed an EXT success rate of 90% without the SBT, compared to 91% for those who performed the SBT, i.e., its use had no impact on EXT outcomes.

This discrepancy in results may be attributed to the fact that the study by Foronda et al.^(25)^ applied a protocol daily to all patients in the test group, unlike the study by Laham et al.,^(21)^ in which there was no standardization for the SBT.

Based on these findings, the protocol developed by Foronda et al.^(25)^ is consistent, and its results stand out in our review.

### Rapid shallow breathing index

The RSBI is the ratio of the respiratory rate (RR) to the TV adjusted by the weight in kg for the pediatric patient (RSBI = (RR/VT)/weight).^(4)^ It is easy to apply and interpret and is one of the most widely used and accepted clinical indices worldwide when evaluating adult patients.^(28,29)^ Tidal volume should be measured during spontaneous breathing for 60 seconds using a respirometer connected to the artificial airway; thus, values < 105 cycles/L predict successful weaning in adults.^(10)^ Its use in pediatrics is not well established because there is no cutoff value that can predict EXT outcomes. The RSBI, included in this study, was evaluated by Khemani et al.,^(22)^ who reported that an increase in RSBI values was associated only with the use of elective or unplanned NIV. However, a cutoff value that defined this increase as a predictor of failure was not presented. For Johnston et al.,^(24)^ values ≥ 6.7 cycles/minute/mL/kg were presented as risk factors for EXT failure; however, the test showed low sensitivity and specificity.

### Maximum inspiratory pressure

The PiMax indicates the strength of the inspiratory muscles and is a simple, noninvasive and easy-to-apply resource. It can be measured by software available in a mechanical ventilator or by manometry.^(30)^ Its evaluation occurs at peak inspiratory pressure, between three and five respiratory cycles, and considers the highest value obtained in the measurements.^(23)^ In the pediatric population, it has been frequently used as a predictor test. In fact, several studies have developed equations for normal PiMax values based on age and sex.^(31-33)^ In intensive care, values < 50cmH_2_O have been associated with EXT failure in the pediatric population.^(15,34)^

Khemani et al.^(22)^ measured PiMax by manometry in pediatric patients; in that study, values ≤ 30cmH_2_O were associated with reintubation. These values were similar to those reported by other studies that defined a cutoff point of < 30cmH_2_O^(33)^ and < 32cmH_2_O^(34)^ as predictors of EXT failure in pediatric patients.

Johnston et al.^(24)^ were the first to evaluate the accuracy of PiMax with a cutoff point of ≤ 50cmH_2_O as a predictor of EXT failure in pediatric patients hospitalized for acute viral bronchiolitis. This measurement was also performed using a pressure gauge, and the results showed high sensitivity and specificity in this population.

### Esophageal pressure/maximal inspiratory pressure

The PI/PiMax is a measure little described in the literature. It is related to the occurrence of respiratory muscle fatigue and is commonly associated with other indices in older studies.^(35,36)^ It was used by Khemani et al.^(22)^ to characterize possible risk factors for pediatric EXT failure. This index showed a strong discrimination power because, when combining the variables respiratory effort, respiratory capacity and respiratory muscle strength over time, the results were related to EXT outcomes.

### Dead space/tidal volume

The measurement of physiological dead space can be used in the management of MV as a prognostic test of lung disease, given that parameters regarding disease severity and pulmonary perfusion are provided.^(37,38)^ However, Riou et al.^(23)^ used it as a predictor of EXT. In this case, a specific device (CO_2_MO Plus Respiratory Profile Monitor) was used to calculate VD/VT from the capnography waveform; values < 0.55 were associated with EXT success in the pediatric population.

### Tension-time index

The tension-time index is an invasive measurement of the diaphragm load and capacity using an esophageal catheter to obtain transdiaphragmatic pressure.^(39)^ According to the American Thoracic Society and the European Respiratory Society,^(40)^ the TTI is an “ideal” physiological variable because it includes relevant measures, such as muscle energy and blood flow. However, the measurement of muscle tension in respiratory pressures is not simple. The TTI can be calculated using the following formula: TTI = (PI/PiMax) x (Tins/Ttot).

Khemani et al.^(22)^ hypothesized that children with respiratory muscle weakness at the time of EXT would be more likely to be reintubated. Thereafter, measures of respiratory load and effort before and after EXT, such as TTI, were used to characterize the possible risk factors. The authors concluded that there was an association between post-EXT TTI and reintubation. However, this association was not observed for the values obtained before extubation.

### Load/force balance

Load/force balance, which assesses the association between the load imposed on the ventilatory system and the ability of the inspiratory muscles to overcome this load, was first described in 2006 by Vassilakopoulos et al.^(41)^ for adult patients. A load/force value = 1 was defined as the cutoff point for successful EXT. This index uses mean airway pressure values during controlled MV and PiMax values and is obtained using the following formula: load/force = 15 × mean airway pressure/PiMax + 0.03 × RSBI - 5.

Johnston et al.^(24)^ were the first to perform load/force measurements to predict EXT failure in pediatric patients and found significantly lower values in the group with successful EXT than in the group with failure, demonstrating that it can be an adequate predictor of EXT failure for pediatric patients because it incorporates both the imposed load and the response of the patient to this load.

### Automated weaning

In recent years, automated weaning strategies have been disseminated. With the modernization of mechanical ventilators, ventilatory support has adapted to the needs of the patient. This safely reduces the duration of MV and delays in weaning.^(42,43)^

Jouvet et al.^(26)^ compared the duration of automated weaning from MV with SmartCare™ PS (DrägerMedical, Lübeck, Germany) *versus* traditional weaning, and their findings corroborated the existing literature, showing a reduction in weaning duration in patients on MV.^(42,43)^ Adaptive support ventilation divides weaning into three phases: respiratory comfort in MV, reduction in PS while maintaining respiratory comfort, and extubation readiness tests at the lowest level of PS.^(43)^

Thus, although it is possible to describe the most commonly used predictive methods in pediatrics, there is no consensus on their applicability in these patients. This is an extremely important subject, but there is heterogeneity in the methodologies applied.

Some limitations of this study should be mentioned; for example, no instrument was used to analyze the quality of the articles. In addition, there were also limitations related to the studies included in this review. The qualitative analysis of the articles showed great heterogeneity in the methodologies used in the studies and in the definitions of EXT failure, in addition to disparities in the data collected, such as age, sex, length of protocol, diagnosis of the children included and variability in the MV devices used, hindering a statistical comparison of the studies.

## CONCLUSION

Based on the findings of this review, the spontaneous breathing test, maximal inspiratory pressure and the rapid shallow breathing index were the predictive methods of choice to determine extubation readiness in pediatric patients. However, there is a lack of standardization of measurements and cutoff points for pediatric patients. Further studies should be conducted in this population using well-defined protocols to elucidate issues raised by the Pediatric Mechanical Ventilation Consensus Conference and thus promote scientific discussion for the standardization of these methods in clinical practice.

## References

[1] Pham T, Brochard LJ, Slutsky AS (2017). Mechanical ventilation: state of the art. Mayo Clin Proc.

[2] Jordan J, Rose L, Dainty KN, Noyes J, Blackwood B (2016). Factors that impact on the use of mechanical ventilation weaning protocols in critically ill adults and children: a qualitative evidence-synthesis. Cochrane Database Syst Rev.

[3] Quintard H, l’Her E, Pottecher J, Adnet F, Constantin JM, De Jong A (2019). Experts’ guidelines of intubation and extubation of the ICU patient of French Society of Anaesthesia and Intensive Care Medicine (SFAR) and Frenchspeaking Intensive Care Society (SRLF): In collaboration with the pediatric Association of French-speaking Anaesthetists and Intensivists (ADARPEF), French-speaking Group of Intensive Care and Paediatric emergencies (GFRUP) and Intensive Care physiotherapy society (SKR). Ann Intensive Care.

[4] Johnston C, Piva JP, Carvalho WB, Garcia PC, Fonseca MC, Hommerding PX (2008). Preditores de falha da extubação em crianças no pós-operatório de cirurgia cardíaca submetidas à ventilação pulmonar mecânica. Rev Bras Ter Intensiva.

[5] Mhanna MJ, Anderson IM, Iyer NP, Baumann A (2014). The use of extubation readiness parameters: a survey of pediatric critical care physicians. Respir Care.

[6] Newth CJ, Venkataraman S, Willson DF, Meert KL, Harrison R, Dean JM, Pollack M, Zimmerman J, Anand KJ, Carcillo JA, Nicholson CE, Eunice Shriver Kennedy National Institute of Child Health and Human Development Collaborative Pediatric Critical Care Research Network (2009). Weaning and extubation readiness in pediatric patients. Pediatr Crit Care Med.

[7] Jaber S, Quintard H, Cinotti R, Asehnoune K, Arnal JM, Guitton C (2018). Risk factors and outcomes for airway failure versus non-airway failure in the intensive care unit: a multicenter observational study of 1514 extubation procedures. Crit Care.

[8] Chawla S, Natarajan G, Shankaran S, Carper B, Brion LP, Keszler M, Carlo WA, Ambalavanan N, Gantz MG, Das A, Finer N, Goldberg RN, Cotten CM, Higgins RD, Eunice Kennedy Shriver National Institute of Child Health and Human Development Neonatal Research Network (2017). Markers of successful extubation in extremely preterm infants, and morbidity after failed extubation. J Pediatr.

[9] Shalish W, Kanbar LJ, Rao S, Robles-Rubio CA, Kovacs L, Chawla S (2017). Prediction of extubation readiness in extremely preterm infants by the automated analysis of cardiorespiratory behavior: study protocol. BMC Pediatr.

[10] Associação de Medicina Intensiva Brasileira (AMIB), Sociedade Brasileira de Pneumologia e Tisiologia (SBPT) (2013). Diretrizes brasileiras de ventilação mecânica 2013.

[11] Nascimento MS, Rebello CM, Vale LA, Santos E, Prado C (2017). Teste de respiração espontânea na previsão de falha de extubação em população pediátrica. Einstein.

[12] Nardi N, Mortamet G, Ducharme-Crevier L, Emeriaud G, Jouvet P (2017). Recent advances in pediatric ventilatory assistance. F1000Res.

[13] Valenzuela J, Araneda P, Cruces P (2014). Weaning from mechanical ventilation in paediatrics. State of the art. Arch Bronconeumol.

[14] Pediatric Acute Lung Injury Consensus Conference Group (2015). Pediatric acute respiratory distress syndrome consensus recommendations from the Pediatric Acute Lung Injury Consensus Conference. Pediatr Crit Care Med.

[15] Faustino EV, Gedeit R, Schwarz AJ, Asaro LA, Wypij D, Curley MA, Randomized Evaluation of Sedation Titration for Respiratory Failure (RESTORE) Study Investigators (2017). Accuracy of an extubation readiness test in predicting successful extubation in children with acute respiratory failure from lower respiratory tract disease. Crit Care Med.

[16] Saikia B, Kumar N, Sreenivas V (2015). Prediction of extubation failure in newborns, infants and children: brief report of a prospective (blinded) cohort study at a tertiary care paediatric centre in India. Springerplus.

[17] Kneyber MC, de Luca D, Calderini E, Jarreau PH, Javouhey E, Lopez-Herce J, Hammer J, Macrae D, Markhorst DG, Medina A, Pons-Odena M, Racca F, Wolf G, Biban P, Brierley J, Rimensberger PC (2017). section Respiratory Failure of the European Society for Paediatric and Neonatal Intensive Care. Recommendations for mechanical ventilation of critically ill children from the Paediatric Mechanical Ventilation Consensus Conference (PEMVECC). Intensive Care Med.

[18] Mendes KD, Silveira RC, Galvão CM (2008). Revisão integrativa: método de pesquisa para a incorporação de evidências na saúde e na enfermagem. Texto Contexto Enferm.

[19] Melnyk BM, Fineout-Overholt E, Melnyk BM, Fineout-Overholt E (2011). Making the case for evidence-based practice. Evidence-based practice in nursing & healthcare: a guide to best practice.

[20] Moher D, Liberati A, Tetzlaff J, Altman DG, PRISMA Group (2009). The PRISMA Group 2009 Preferred reporting items for systematic reviews and meta-analyses: the PRISMA statement. PLoS Med.

[21] Laham JL, Breheny PJ, Rush A (2015). Do clinical parameters predict first planned extubation outcome in the pediatric intensive care unit?. J Intensive Care Med.

[22] Khemani RG, Sekayan T, Hotz J, Flink RC, Rafferty GF, Iyer N (2017). Risk factors for pediatric extubation failure: the importance of respiratory muscle strength. Crit Care Med.

[23] Riou Y, Chaari W, Leteurtre S, Leclerc F (2012). Predictive value of the physiological deadspace/tidal volume ratio in the weaning process of mechanical ventilation in children. J Pediatr (Rio J).

[24] Johnston C, de Carvalho WB, Piva J, Garcia PC, Fonseca MC (2010). Risk factors for extubation failure in infants with severe acute bronchiolitis. Resp Care.

[25] Foronda FK, Troster EJ, Farias JA, Barbas CS, Ferraro AA, Faria LS (2011). The impact of daily evaluation and spontaneous breathing test on the duration of pediatric mechanical ventilation: a randomized controlled trial. Crit Care Med.

[26] Jouvet PA, Payen V, Gauvin F, Emeriaud G, Lacroix J (2013). Weaning children from mechanical ventilation with a computer-driven protocol: a pilot trial. Intensive Care Med.

[27] Leclerc F, Noizet O, Botte A, Binoche A, Chaari W, Sadik A (2010). [Weaning from invasive mechanical ventilation in pediatric patients (excluding premature neonates)]. Arch Pediatr.

[28] Nemer SN, Barbas CS (2011). Parâmetros preditivos para o desmame da ventilação mecânica. J Bras Pneumol.

[29] Souza LC, Lugon JR (2015). Índice de respiração rápida e superficial como previsor de sucesso de desmame da ventilação mecânica: utilidade clínica quando mensurado a partir de dados do ventilador. J Bras Pneumol.

[30] Cox DW, Verheggen MM, Stick SM, Hall GL (2012). Characterization of maximal respiratory pressures in health children. Respiration.

[31] Gomes EL, Peixoto-Souza FS, Carvalho EF, Nascimento ES, Sampaio LM, Eloi JS (2014). Maximum respiratory pressures: values found and predicted in children. J Lung Pulm Respir Res.

[32] Mendes RF, Campos TF, Macêdo TM, Borja RO, Parreira VF, Mendonça KM (2013). Prediction equations for maximal respiratory pressures of Brazilian adolescents. Braz J Phys Ther.

[33] Heinzmann-Filho JP, Vasconcellos Vidal PC, Jones MH, Donadio MV (2012). Normal values for respiratory muscle strength in healthy preschoolers and school children. Respir Med.

[34] Harikumar G, Egberongbe Y, Nadel S, Wheatley E, Moxham J, Greenough A (2009). Tension-time index as a predictor of extubation outcome in ventilated children. Am J Respir Crit Care Med.

[35] Yang KL (1993). Inspiratory pressure/maximal inspiratory pressure ratio: a predictive index of weaning outcome. Intensive Care Med.

[36] Hahn A, Ankermann T, Claass A, Mann M, Lindemann H, Neubauer BA (2008). Non-invasive tension time index in relation to severity of disease in children with cystic fibrosis. Pediatr Pulmonol.

[37] Bourgoin P, Baudin F, Brossier D, Emeriaud G, Wysocki M, Jouvet P (2017). Assessment of Bohr and Enghoff dead space equations in mechanically ventilated children. Respir Care.

[38] Bhalla AK, Rubin S, Newth CJ, Ross P, Morzov R, Soto-Campos G (2015). Monitoring dead space in mechanically ventilated children: volumetric capnography versus time-based capnography. Respir Care.

[39] Currie A, Patel DS, Rafferty GF, Greenough A (2011). Prediction of extubation outcome in infants using the tension time index. Arch Dis Child Fetal Neonatal Ed.

[40] American Thoracic Society/European Respiratory Society (2002). ATS/ERS Statement on respiratory muscle testing. Am J Respir Crit Care Med.

[41] Vassilakopoulos T, Routsi C, Sotiropoulou C, Bitsakou C, Stanopoulos I, Roussos C (2006). The combination of the load/force balance and the frequency/tidal volume can predict weaning outcome. Intensive Care Med.

[42] Aguilar Arzápalo MF, Escalante Castillo AE, Góngora Mukul JJ, López Avendaño VG, Cetina Cámara MA, Magdaleno Lara GA (2016). Eficacia del protocolo automático de destete (SmartCare®) comparado con protocolos no automatizados en la desconexión de la ventilación mecánica en pacientes adultos de la unidad de cuidados intensivos. Rev Asoc Mex Med Crít Ter Intensiva.

[43] Rose L, Schultz MJ, Cardwell CR, Jouvet P, McAuley DF, Blackwood B (2015). Automated versus non-automated weaning for reducing the duration of mechanical ventilation for critically ill adults and children: a cochrane systematic review and meta-analysis. Crit Care.

