# Supplementary material for: Extubation in the pediatric intensive care unit: predictive methods. An integrative literature review
Source: Rev Bras Ter Intensiva. 2021 Apr-Jun;33(2):304–11. doi: 10.5935/0103-507X.20210039 (PMC8275073; doi:10.5935/0103-507X.20210039)
Supplement: Supplementary file 1 [file rbti-33-02-0304-suppl01.pdf]

Extubation in the pediatric intensive care unit: predictive methods. An integrative literature review

Extubação em unidade de terapia intensiva pediátrica: métodos preditores. Uma revisão integrativa da literatura

Jéssica Cristina da Silva Moura[1], Lívea Gianfrancesco[1], Tiago Henrique de Souza[1], Taís Daiene Russo Hortencio[1,2], Roberto José Negrão Nogueira[1,2]

Table 1S - The complete search terms list used for PubMed®, *Biblioteca Virtual em Saúde* and Cochrane

((((*Desmame do Respirador*) OR *Retirada do Respirador*) OR *Retirada do Ventilador*) AND *Respiração Artificial*) AND *Extubação*) AND *Pediatria*
((((*Desmame do Respirador*) OR *Retirada do Respirador*) OR *Retirada do Ventilador*) AND *ventilação mecânica*) AND Extubação) AND *Pediatria*
(((*Desmame do Respirador mecânico*) AND *ventilação mecânica*) AND *Extubação*) AND *Pediatria*
((((((((((Ventilator Weaning) OR Weaning, Respirator) OR Weaning, Ventilator) OR Respirator Weaning) AND Respiration, Artificial) OR Artificial Respirations) OR Respirations, Artificial) AND Airway Extubation) OR Airway Extubations) OR Extubation, Airway) OR Extubations, Airway) AND Pediatrics
((((((((((Ventilator Weaning) OR Weaning, Respirator) OR Weaning, Ventilator) OR Respirator Weaning) AND Mechanical Ventilation) OR Mechanical Ventilations) OR Ventilations, Mechanical) AND Airway Extubation) OR Airway Extubations) OR Extubation, Airway) OR Extubations, Airway) AND Pediatrics
(((((((((((Ventilator Weaning) OR Weaning, Respirator) OR Weaning, Ventilator) OR Respirator Weaning) AND Respiration, Artificial) OR Artificial Respirations) OR artificial Respiration) OR Respirations, Artificial) AND Endotracheal Extubation) OR Extubation, Endotracheal) OR Endotracheal Extubations) OR Extubations, Endotracheal) AND Pediatrics
(((((((((((Ventilator Weaning) OR Weaning, Respirator) OR Weaning, Ventilator) OR Respirator Weaning) AND Respiration, Artificial) OR Artificial Respirations) OR artificial Respiration) OR Respirations, Artificial) AND Intratracheal Extubation) OR Intratracheal Extubations) OR Extubations, Intratracheal) OR Extubation, Intratracheal) AND Pediatrics
(((((((((((Ventilator Weaning) OR Weaning, Respirator) OR Weaning, Ventilator) OR Respirator Weaning) AND Respiration, Artificial) OR Artificial Respirations) OR artificial Respiration) OR Respirations, Artificial) AND Tracheal Extubation) OR Tracheal Extubations) OR Extubation, Tracheal) OR Extubations, Tracheal) AND Pediatrics
(((((((((((Ventilator Weaning) OR Weaning, Respirator) OR Weaning, Ventilator) OR Respirator Weaning) AND Mechanical Ventilation) OR Mechanical Ventilations) OR Ventilations, Mechanical) OR Ventilation, Mechanical) AND Endotracheal Extubation) OR Endotracheal Extubations) OR Extubation, Endotracheal) OR Extubations, endotracheal) AND Pediatrics
(((((((((((Ventilator Weaning) OR Weaning, Respirator) OR Weaning, Ventilator) OR Respirator Weaning) AND Mechanical Ventilation) OR Mechanical Ventilations) OR Ventilations, Mechanical) OR Ventilation, Mechanical) AND Intratracheal Extubation) OR intratracheal Extubations) OR Extubation, intratracheal) OR Extubations, intratracheal) AND Pediatrics
(((((((((((Ventilator Weaning) OR Weaning, Respirator) OR Weaning, Ventilator) OR Respirator Weaning) AND Mechanical Ventilation) OR Mechanical Ventilations) OR Ventilations, Mechanical) OR Ventilation, Mechanical) AND tracheal Extubation) OR tracheal Extubations) OR Extubation, tracheal) OR Extubations, tracheal) AND Pediatrics
((((((((((Ventilator Weaning, Mechanical) OR Weaning, Mechanical Ventilator) OR Mechanical Ventilator Weaning) AND Respiration, Artificial) OR Artificial Respirations) OR Artificial Respiration) OR Respirations, Artificial) AND Airway Extubation) OR Airway Extubations) OR Extubation, Airway) OR Extubations, Airway) AND Pediatrics
((((((((((Ventilator Weaning, Mechanical) OR Weaning, Mechanical Ventilator) OR Mechanical Ventilator Weaning) AND Respiration, Artificial) OR Artificial Respirations) OR Artificial Respiration) OR Respirations, Artificial) AND Endotracheal Extubation) OR Endotracheal Extubations) OR Extubation, Endotracheal) OR Extubations, Endotracheal) AND Pediatrics
((((((((((Ventilator Weaning, Mechanical) OR Weaning, Mechanical Ventilator) OR Mechanical Ventilator Weaning) AND Respiration, Artificial) OR Artificial Respirations) OR Artificial Respiration) OR Respirations, Artificial) AND intratracheal Extubation) OR intratracheal Extubations) OR Extubation, intratracheal) OR Extubations, intratracheal) AND Pediatrics
((((((((((Ventilator Weaning, Mechanical) OR Weaning, Mechanical Ventilator) OR Mechanical Ventilator Weaning) AND Respiration, Artificial) OR Artificial Respirations) OR Artificial Respiration) OR Respirations, Artificial) AND tracheal Extubation) OR tracheal Extubations) OR Extubation, tracheal) OR Extubations, tracheal) AND Pediatrics
((((((((((Ventilator Weaning, Mechanical) OR Weaning, Mechanical Ventilator) OR Mechanical Ventilator Weaning) AND Mechanical Ventilation) OR Mechanical Ventilations) OR Ventilations, Mechanical) OR Ventilation, Mechanical) AND Airway Extubation) OR Airway Extubations) OR Extubation, Airway) OR Extubations, Airway) AND pediatrics
((((((((((Ventilator Weaning, Mechanical) OR Weaning, Mechanical Ventilator) OR Mechanical Ventilator Weaning) AND Mechanical Ventilation) OR Mechanical Ventilations) OR Ventilations, Mechanical) OR Ventilation, Mechanical) AND Endotracheal Extubation) OR Endotracheal Extubations) OR Extubation, Endotracheal) OR Extubations, Endotracheal) AND pediatrics
((((((((((Ventilator Weaning, Mechanical) OR Weaning, Mechanical Ventilator) OR Mechanical Ventilator Weaning) AND Mechanical Ventilation) OR Mechanical Ventilations) OR Ventilations, Mechanical) OR Ventilation, Mechanical) AND intratracheal Extubation) OR intratracheal Extubations) OR Extubation, intratracheal) OR Extubations, intratracheal) AND pediatrics
((((((((((Ventilator Weaning, Mechanical) OR Weaning, Mechanical Ventilator) OR Mechanical Ventilator Weaning) AND Mechanical Ventilation) OR Mechanical Ventilations) OR Ventilations, Mechanical) OR Ventilation, Mechanical) AND tracheal Extubation) OR tracheal Extubations) OR Extubation, tracheal) OR Extubations, tracheal) AND pediatrics

Table 2S - The complete search terms list used for Scopus

(ALL ("Ventilator Weaning")) AND (ALL ("respiration artificial")) AND (ALL ("airway extubation")) AND (ALL (pediatrics))
(ALL ("Ventilator Weaning")) OR (ALL ("respirator weaning")) AND (ALL ("respiration artificial")) OR (ALL ("Artificial Respiration")) AND (ALL ("airway extubation")) OR (ALL ("extubation, airway")) AND (ALL (pediatrics))
(ALL ("Ventilator Weaning")) OR (ALL ("respirator weaning")) AND (ALL ("mechanical ventilation")) OR (ALL ("ventilation, mechanical")) AND (ALL ("airway extubation")) OR (ALL ("extubation, airway")) AND (ALL (pediatrics))
(ALL ("Ventilator Weaning")) OR (ALL ("respirator weaning")) AND (ALL ("respiration artificial")) OR (ALL ("Artificial Respiration")) AND (ALL ("endotracheal extubation")) OR (ALL ("endotracheal extubations")) AND (ALL (pediatrics))
(ALL ("Ventilator Weaning")) OR (ALL ("respirator weaning")) AND (ALL ("respiration artificial")) OR (ALL ("Artificial Respiration")) AND (ALL ("intratracheal extubation")) OR (ALL ("extubation, intratracheal")) AND (ALL (pediatrics))
(ALL ("Ventilator Weaning")) OR (ALL ("respirator weaning")) AND (ALL ("respiration artificial")) OR (ALL ("Artificial Respiration")) AND (ALL ("tracheal extubations")) OR (ALL ("tracheal extubation")) AND (ALL (pediatrics))
(ALL ("Ventilator Weaning")) OR (ALL ("respirator weaning")) AND (ALL ("mechanical ventilation")) OR (ALL ("ventilation, mechanical")) AND (ALL ("endotracheal extubation")) OR (ALL ("endotracheal extubations")) AND (ALL (pediatrics))
(ALL ("Ventilator Weaning")) OR (ALL ("respirator weaning")) AND (ALL ("mechanical ventilation")) OR (ALL ("ventilation, mechanical")) AND (ALL ("tracheal extubation")) OR (ALL ("tracheal extubations")) AND (ALL (pediatrics))
(ALL ("Ventilator Weaning")) OR (ALL ("respirator weaning")) AND (ALL ("mechanical ventilation")) OR (ALL ("ventilation, mechanical")) AND (ALL ("extubation, intratracheal")) OR ALL ("intratracheal extubation")) AND (ALL (pediatrics))
